# On the origin of prokaryotic "species": the taxonomy of halophilic *Archaea*

**DOI:** 10.1186/1746-1448-4-5

**Published:** 2008-05-16

**Authors:** Priya DasSarma, Shiladitya DasSarma

**Affiliations:** 1 University of Maryland Biotechnology Institute, Center of Marine Biotechnology, 701 East Pratt Street, Baltimore, MD 21202, USA

## Abstract

The consistent use of the taxonomic system of binomial nomenclature (genus and species) was first popularized by Linnaeus nearly three-hundred years ago to classify mainly plants and animals. His main goal was to give labels that would ensure that biologists could agree on which organism was under investigation. One-hundred fifty years later, Darwin considered the term species as one of convenience and not essentially different from variety. In the modern era, exploration of the world's niches together with advances in genomics have expanded the number of named species to over 1.8 million, including many microorganisms. However, even this large number excludes over 90% of microorganisms that have yet to be cultured or classified. In naming new isolates in the microbial world, the challenge remains the lack of a universally held and evenly applied standard for a species. The definition of species based on the capacity to form fertile offspring is not applicable to microorganisms and 70% DNA-DNA hybridization appears rather crude in light of the many completed genome sequences. The popular phylogenetic marker, 16S rRNA, is tricky for classification since it does not provide multiple characteristics or phenotypes used classically for this purpose. Using most criteria, agreement may usually be found at the genus level, but species level distinctions are problematic. These observations lend credence to the proposal that the species concept is flawed when applied to prokaryotes. In order to address this topic, we have examined the taxonomy of extremely halophilic *Archaea*, where the order, family, and even a genus designation have become obsolete, and the naming and renaming of certain species has led to much confusion in the scientific community.

## Historical background

An important challenge in the classification of microorganisms is ensuring that scientists can follow the pedigree of isolates in the literature. This laudable goal is however especially difficult to achieve for microbes that have a long history and where variants have been isolated and re-isolated from an abundance of niches in the laboratories of many different investigators. For example, the group of extremely salt-loving halophilic microbes which produce red, pink, and purple hues in hypersaline ponds used to make salt from the sea, were among the earliest microorganisms to be recognized and described. They were, not surprisingly, originally identified as agents of food spoilage, before the advent of refrigeration when salting was widely used for preserving fish and meats [1]. Some early isolates from dried and salted codfish (Klippfisch) were documented in a 1919 review on causative agents of fish reddening by the German botanist, Klebahn [2]. Notably, he isolated and named "*Bacillus halobius ruber*", aware that these bright vermillion halophilic microbes were not spore formers, but his isolate was subsequently lost. A dozen years later, halophilic isolates thought to be similar to Klebahn's, were named "*Bacterium halobium*" by Petter in the Kluyver laboratory in Delft, Holland [3]. In the 1940s–1960s, additional halophilic microorganisms were isolated from different countries and reported in the scientific literature with names such as *Halobacterium halobium, H. salinarium*, and *H. cutirubrum *[4]. Many of these isolates were deposited in US, Canadian, and European culture collections, but a substantial number of them were subsequently lost or renamed. Revision of the taxonomy of these extremely halophilic genera from *Bacillus *to *Bacterium *to *Halobacterium *reflected the increasing sophistication of our knowledge of the microbial world during this period, and these changes were generally accepted by the scientific community. In the modern era, there have been many more proposals for taxonomic revisions among these halophiles, some of which have been readily acceptable, and others that have since been challenged or refuted [5,6].

## A proposal to modernize haloarchaeal taxonomy and terminology

Among extremely halophilic microorganisms, the distinction of halophilic *Archaea *from halophilic *Bacteria *became apparent in the 1970's through the molecular phylogenetic work of Woese, who proposed the three-domain view of life. While halophilic microorganisms represented many different taxonomic groups in the bacterial domain, those in the archaeal domain fell into a single order (*Halobacteriales*) and family (*Halobacteriacae*) [7]. Our understanding of the existence of the three domains has created ambiguity in the terminology used, since 'halobacteria' traditionally referred to all extremely halophilic microorganisms, including both halophilic *Bacteria *and halophilic *Archaea*. In order to clarify the definitions, we propose that the term halobacteria be reserved only for halophiles that are members of the bacterial domain, while haloarchaea be used only for halophiles that are members of the archaeal domain. In addition, on a taxonomic level, the order *Halobacteriales *should be designated as *Haloarchaeales *and the family *Halobacteriaceae *should be as *Haloarchaeaceae*. Finally, the *Halobacterium *genus would be better named *Haloarchaeum *to reflect its membership in the archaeal rather than the bacterial domain. These revisions would help update the taxonomy and terminology of halophilic microorganisms to be consistent with our current understanding of the microbial world.

## Taxonomic ambiguity among species

While our proposed revision of haloarchaeal taxonomy is relatively simple, disentangling the taxonomy and pedigree within the original genus, *Halobacterium *(*Haloarchaeum*), is considerably more complex. Over the past twenty-five to fifty years, this genus witnessed a contraction in the number of recognized species from over a dozen to just a single species. While some acquired new genus designations (e.g. *Halobacterium volcanii *changed to *Haloferax volcanii *and *Halobacterium marismortui *to *Haloarcula marismortui*), in 1990, Grant and Larsen proposed combining three common species, *Halobacterium halobium*, *H. salinarium*, and *H. cutirubrum*, into a single one, *H. salinarium *[7]. However, this proposal was not fully accepted by the community since the changes were not fully in accordance with the rules of the Bacteriological Code [8]. In particular, *halobium *predated *salinarium *in the literature and, by convention, the former name should have taken precedence over the latter. To complicate matters further, in 1996, Ventosa and Oren proposed renaming of *H. salinarium *to *H. salinarum *[9], removing an "*i*", in their opinion, for linguistic reasons. However, many investigators dissented and continued to use the original species designations. In Euzéby's *List of Prokaryotic Names with Standing in Nomenclature *[10], he reported that *salinarium*, is derived from the Latin adjective *salinarius a um*, meaning "of salt works", while *salinarum *is derived from *salinae arum*, meaning "salt works", and concluded that *salinarium *was indeed correct. There is no doubt that naming and renaming of these species has left the taxonomy of *Halobacterium *(*Haloarchaeum*) species in disarray in the literature and in the haloarchaeal community.

Perhaps the worst case of taxonomic ambiguity is for the first *Halobacterium *(*Haloarchaeum*) isolate sequenced and also the most widely used haloarchaeal strain, which was published under the name *H. halobium *strain NRC-1 [11]. The origin of strain NRC-1 is uncertain, though it likely appeared from the 1960's collection of W. Stoeckenius and was disseminated via W.F. Doolittle (personal communications) to S. DasSarma in the 1980's. In 2000, the NRC-1 strain was deposited by the DasSarma laboratory in the American Type Culture Collection (ATCC no. 700922) for standardization and distribution in the research community and has since been used by Carolina Biological Supply Company in the educational sphere (Carolina no. 154777) [12]. Stocks of the original culture used for sequencing are also maintained in the DasSarma laboratory. As a result of uncertainties regarding the origin of this strain, the authors of the complete genome sequencing paper [11] dropped the species designation, reverting to "*Halobacterium *sp. strain NRC-1", while its pedigree was being rigorously established and the relationships within this group of organisms fully clarified. However, despite the lack of appearance of definitive information on the identity of NRC-1, Gruber et al. [13] published a paper in 2004 reclassifying the wild-type isolate as a strain of the '*H. salinarum*' species. In so doing, these authors ignored a variety of differences between strains, including their own pulsed-field gel patterns, as well as variations in restriction maps of the unstable resident megaplasmids. Overreliance on phylogenetic trees based on 16S rRNA sequences, some of which are already known to be divergent even within single haloarchaeal species [14], was a serious shortcoming of this study.

**Figure 1:**
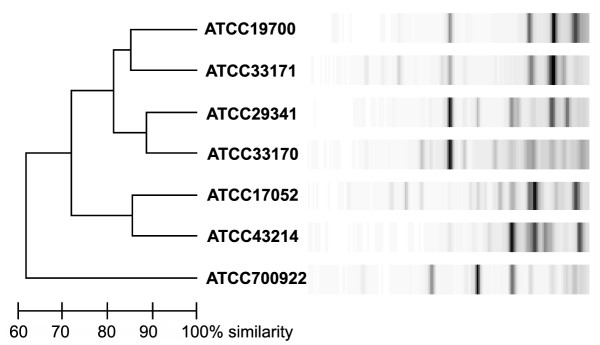
Genotyping of *Halobacterium *(*Haloarchaeum*) isolates using the DiversiLab repPCR system by Cleland et al. at the American Type Culture Collection [16]. The two sequenced *Halobacterium *species are included, the model strain NRC-1 (ATCC 700922) and strain R-1 (ATCC 29341) [14], which appear to differ sufficiently enough to warrant distinct species designations.

Reexamination of the available data on *Halobacterium *(*Haloarchaeum*) isolates at the phylogenetic and taxonomic levels confirms the existence of serious complications. The 16S rRNA sequences vary in nearly all of the originally distinct species, with that of NRC-1 and *H. salinarium *differing in several positions. Differences also exist between the NRC-1 and *H. halobium *23S rRNA sequences, as well as between NRC-1 and *H. cutirubrum *5S rRNA. In fact, a recent publication even reported a major deletion in the 16S rRNA promoter region of '*H. salinarum*' (originally *H. halobium*) strain R-1 in comparison to strain NRC-1 and other similar strains [15]. The most compelling case for the existence of substantial taxonomic diversity among *Halobacterium *(*Haloarchaeum*) isolates is from the recent genotyping analysis of Cleland et al. using the DiversiLab repPCR system [16]. In this study, some of the seven *Halobacterium *(*Haloarchaeum*) strains in the ATCC collection show differences quantitatively similar to haloarchaea that are classified as different genera of the *Halobacteriaceae *(*Haloarchaeaceae*) family. Their examination of NRC-1 by this method (Figure 1) showed that this strain fell below 70% similarity compared to other '*H. salinarium' *strains in ATCC, suggesting that NRC-1 should be given an entirely new species designation using that criterion. This analysis supports our viewpoint that it is premature to reclassify all of these *Halobacterium *(*Haloarchaeum*) isolates as a single species, especially without an existing consensus in the community on the definition of what constitutes a "species" among these organisms.

## Phenotypes and pedigree

At the phenotypic level, *Halobacterium *(*Haloarchaeum*) strains have some clear-cut differences. An especially striking difference is the absence, in '*H. salinarum' *(e.g. strain R-1), of gas vesicles, which are characteristic of *Halobacterium *sp. NRC-1. Gas vesicles permit NRC-1 to move vertically in the water column in response to oxygen, light, and temperature, and the corresponding expression of gas vesicle protein genes can be clearly seen in DNA microarray experiments [17,18]. The lack of gas vesicles in '*H. salinarum*' indicates that these organisms exist in significantly different environments from NRC-1, with the latter inhabiting dynamic ones, and the former in more constant environments. This notable phenotypic difference is easily visible even to the naked eye. Some other *Halobacterium *(*Haloarchaeum*) species contain two different morphological types of gas vesicles in the same cell, those which are narrower and diagnostic of species inhabiting deep habitats, as well as those which are wider and found in shallow brines (like NRC-1), suggesting the widespread environmental distribution of these species. Examined more broadly, isolate-specific differences have been shown to exist at the level of antibiotic-resistance markers, measured cations in cells, and protein, lipid, and sugar content in the cell envelopes [4].

The pedigree of the various strains of *Halobacterium *(*Haloarchaeum*) being studied in laboratories worldwide is also confounding. One example is the unclear relationship between NRC-1 and R-1, and another similar strain S-9, a purple membrane overproducer. While some investigators reported that NRC-1, R-1, and S-9, were very close relatives [19], others indicated otherwise [20,21]. The stable gas vesicle-deficient strain, R-1, may be the parent of S-9, a strain which was isolated after extensive chemical mutagenesis, but R-1 is probably not a descendent of NRC-1. Not surprisingly, the genome sequencing results showed at least 200 kb of additional DNA in R-1 compared to NRC-1 and very little similarity in their resident megaplasmids [15]. Many additional examples of incongruent taxonomy and pedigree among *Halobacterium *(*Haloarchaeum*) species are reviewed by Grant and Larsen [7] and Tindall [8]. Clearly, the frequent and questionable revisions in the taxonomy of these interesting microbes, and at times, the lack of careful maintenance and documentation of their pedigree, are a serious impediment to advancing the field.

## Epilogue

The incredible precision of the genomic era has empowered microbiologists with the genetic blueprints of more than a thousand microorganisms and allowed for the development of many new approaches for the interrogation of their biology. Unfortunately, studies of certain microbes, such as the haloarchaea, have been made exceedingly difficult by the arbitrary and unnecessary renaming of strains, poor record keeping of pedigree, and the lack of a universal definition of species. All of these shortcomings make it likely that future generations will not be able to fully interpret and utilize the current literature, ultimately diminishing the contributions of both past and present generations. While rigorous genetic studies and complete genome sequences are destined to make a permanent contribution to the field, taxonomic rearrangements based on inadequate experimentation and flawed logic hold it back. Although these points resonate especially true among the haloarchaea, we suspect that similar taxonomic issues and challenges are quite widespread among other prokaryotes as well and are deserving of further scrutiny.

## Competing interests

The authors declare that they have no competing interests.

## Authors' contributions

Both authors contributed to the development of the concepts and writing of the manuscript.
